# In normal aging ventricular system never attains pathological values of Evans' index

**DOI:** 10.18632/oncotarget.7644

**Published:** 2016-02-23

**Authors:** Paolo Missori, Aurelia Rughetti, Simone Peschillo, Gianfranco Gualdi, Claudio Di Biasi, Italo Nofroni, Lucio Marinelli, Francesco Fattapposta, Antonio Currà

**Affiliations:** ^1^ Department of Neurology and Psychiatry, Neurosurgery, Policlinico Umberto I, ‘Sapienza’ University of Rome, Rome, Italy; ^2^ Department of Experimental Medicine, ‘Sapienza’ University of Rome, Rome, Italy; ^3^ Department of Neurology and Psychiatry, Endovascular Neurosurgery/Interventional Neuroradiology, ‘Sapienza’ University of Rome, Rome, Italy; ^4^ Department of Radiology, Policlinico Umberto I, ‘Sapienza’ University of Rome, Rome, Italy; ^5^ Department of Public Health and Infectious Diseases, Medical Statistics and Biometry, ‘Sapienza’ University of Rome, Rome, Italy; ^6^ Department of Neuroscience, Rehabilitation, Ophthalmology, Genetics, Maternal and Child Health, University of Genova, Genova, Italy; ^7^ Department of Medico-Surgical Sciences and Biotechnologies, Neurology, A. Fiorini Hospital, Terracina, LT, ‘Sapienza’ University of Rome, Polo Pontino, Italy

**Keywords:** aging, brain, enlargement, Evans' index, ventricular system, Gerotarget

## Abstract

Ventricular enlargement in normal aging frequently forces the radiological diagnosis of hydrocephalus, but the reliability of Evans' index as a radiological marker of abnormal ventricular enlargement (values > 0.30) during aging is not assessed. Here we analyze ventricular size during aging and the reliability of Evans' index as a radiological marker of abnormal ventricular enlargement. We calculated Evans' index in the axial Computed Tomography scans of 1221 consecutive individuals (aged 45-101 years) from an emergency department. Stratified analysis of one-year cohorts showed that the mean Evans' index value per class was invariably < 0.30. Roughly one out five Computed Tomography scans was associated with Evans' index values > 0.30 and Evans' index values increased with age. The risk of having an Evans' index value > 0.30 increased by 7.8% per year of age (*p* < 0.001) and males were at 83.9% greater risk than females (*p* < 0.001). Overall, this study shows that normal aging enlarges the ventricular system, but never causes abnormal ventricular enlargement. Evans' index values > 0.30 should reflect an underlying neurological condition in every individual.

## INTRODUCTION

Current guidelines suggest the use of Evans’ index [EI] as a marker for radiological diagnosis of hydrocephalus [1, 2]. EI is based on pneumoencephalographic findings for pediatric hydrocephalus reported in the early 1940s [3], and is calculated by dividing the maximum width of the frontal horns by the maximum width of the inner table of the cranium at the level of Monro's foramens in the frontal horns. EI values >0.30 are considered indicative of hydrocephalus. Subsequent studies employing CT confirmed the diagnostic reliability of EI [4, 5].

Advanced neuroimaging techniques indicate that measuring ventricular volume is the best strategy for evaluating hydrocephalus [6, 7]. Unfortunately, this measure is complex and is not readily available in most resource-limited settings, thereby being of little use for daily, routine quantitation of ventricular size.

An easy-to-use, accessible, cost-effective, reliable tool for defining ventricular size in normal aging or neurological conditions would be extremely useful in clinical practice. We therefore designed this transversal radiological study to assess the reliability of Evans’ index (EI) as a radiological marker of abnormal ventricular enlargement (values >0.30) during aging, independently from eventually associated neurological conditions, in a large series of consecutive head scans from an emergency department (ED).

## RESULTS

**Figure 1:**
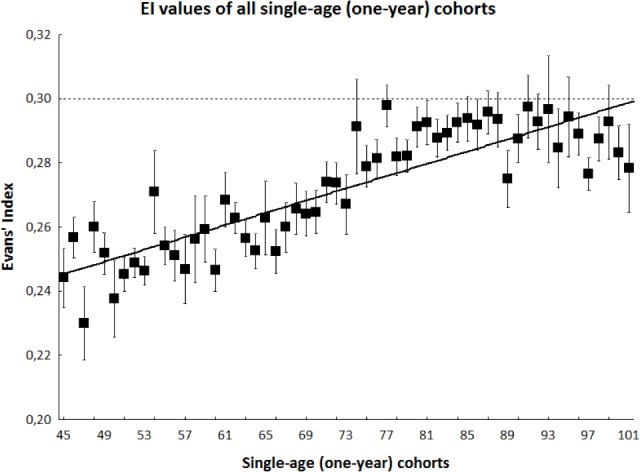
Black squares and bars indicate mean ± SE values of EI in each single-age (one-year) cohort Continuous line indicates linear regression. Dashed line indicates the cut-off value of EI.

Out of 1650 examined CT scans, 1221 were eligible for EI calculation (mean±SD EI value, 0.27±0.04). Scans came from 656 women (53.7%, aged 75.5±15.0 years) and 565 men (46.3%, aged 71.3±13.2 years; entire study population, 73.6±14.4 years). EI was significantly greater in men (0.28±0.04) than in women (0.27±0.04; p<0.01, Student's *t*-test). EI increased with age in a similar fashion in both sexes (linear regression coefficient = 0.001, p<0.001; Figure 1). Kappa value of 0.86 indicated “almost perfect agreement” between observers in calculating EI.

EI values >0.30 were calculated for 236 scans (19.3%; EI 0.33±0.02; 121 men [21.4%], 115 women [17.5%]; p=0.036, chi-squared test). Multiple logistic regression analysis demonstrated that the risk of having an EI value >0.30 increased by 7.8% per year of age (p<0.001) and that males were at 83.9% greater risk than females (p<0.001). Stratified analysis of one-year cohorts showed that the mean EI value per class was invariably <0.30 (Figure 1).

## DISCUSSION

This transversal radiological study of consecutive ED head CT scans provides evidence that roughly one out five CT scans is associated with EI values >0.30 and that EI values increase with age. Here, the mean EI values of all one-year cohorts were <0.30. Therefore an EI value ≥ 0.30 denotes an abnormally enlarged ventricular system, although it does not specify whether the enlargement relates to loss of brain volume (i.e. atrophy) or disturbance in cerebrospinal fluid dynamics.

In healthy elderly individuals, neuroimaging often shows evidence of both brain atrophy and ventricular enlargement [8-12]. When ventricular enlargement is measured through Evans’ index, MRI studies on normal elderly subjects show an EI >0.30 from 2.8 to 17% of scans [7, 13].

The rate of EI values >0.30 determined here likely reflects at least two concurrent factors. First, an age-based selection bias excluded scans from subjects younger than 45 years, who possess notoriously small ventricles. Second, ED-based enrollment may have caused subjects prone to falls to be over-represented, leading to the over-representation in our dataset of neurological conditions favoring frequent falling and head injury.

The observed increase in EI values with age indicates that aging per se induces progressive ventricular enlargement. However, stratified analysis indicated that mean EI values in all one-year cohorts were <0.30 (Figure 1). Thus, aging enlarges the ventricular system, but normally below the threshold for abnormal ventricular enlargement. On the other hand, neurological conditions leading to loss of brain volume or disturbance in cerebrospinal fluid dynamics become more and more frequent with increasing age. Therefore, we suggest that whatever the age of the patient, an EI >0.30 should prompt the physician to search for degenerative diseases such as Alzheimer's disease or other primary dementias, Parkinson's disease or other parkinsonisms, vascular diseases such as vascular cognitive, executive or gait impairment, and other conditions leading to the adult chronic hydrocephalus. The present study aimed simply to test EI as a radiological marker of abnormal ventricular enlargement during aging, independently from the eventually associated neurological conditions. The very high frequency of EI values >0.30 found here reflects over-representation in our ED-based series of neurological conditions related to loss of brain volume, obviously more frequent as age grows. The almost doubled risk of having an EI value >0.30 in men a brief comment, i.e. it may reflect a gender difference on brain size, or suggest that being male may favour abnormal ventricular enlargement.

In conclusion, this transversal study demonstrates that EI is a reliable tool for longitudinal assessment of ventricular size in normal aging, and confirms that >0.30 is a valid, age-independent cut-off for disclosing abnormal ventricular enlargement. EI >0.30 should be considered a red flag, motivating clinical and neuroimaging monitoring to identify the underlying neurological condition.

## MATERIALS AND METHODS

A retrospective review, IRB-approved (ref. 3601 march 26 2015) of head CT data was conducted from all individuals (aged 45-101 years) who were admitted to our ED between June and December 2009. All scans were part of early work-up for acute neurological symptoms or head injury. We excluded from analysis all scans showing hemorrhagic strokes, neoplasms, or post-traumatic hemorrhages. Two observers (one neuroradiologist, one neurosurgeon) calculated the EI in the axial scans at the level of Monro's foramens. Agreement between observers was evaluated using Kappa statistic. Student's t-test, chi-squared test, and multiple logistic regression analysis of EI values were obtained from all scans, one-year [single-age] cohorts, and both sexes. Statistical significance was set at p<0.001.
